# Serum levels of Vanin-2 increase with obesity in relation to inflammation of adipose tissue and may be a predictor of bariatric surgery outcomes

**DOI:** 10.3389/fnut.2023.1270435

**Published:** 2023-12-14

**Authors:** Shan Geng, Dongmei Chen, Yanping Wang, Xingrui Yu, Dan Zuo, Xinlu Lv, Xuelian Zhou, Chengju Hu, Xuesong Yang, Xujue Ma, Wenjing Hu, Jiazhuang Xi, Shaohong Yu

**Affiliations:** ^1^The Affiliated Dazu Hospital of Chongqing Medical University, Chongqing, China; ^2^State Key Laboratory of Ultrasound in Medicine and Engineering, College of Biomedical Engineering, Chongqing Medical University, Chongqing, China; ^3^Department of Otorhinolaryngology, The Affiliated Dazu Hospital of Chongqing Medical University, Chongqing, China; ^4^Institute of Information, Xiamen University, Xiamen, China; ^5^Department of Clinical Nutrition, The Affiliated Dazu Hospital of Chongqing Medical University, Chongqing, China; ^6^Department of Endocrinology, The Affiliated Dazu Hospital of Chongqing Medical University, Chongqing, China; ^7^Department of General Surgery, The Affiliated Dazu Hospital of Chongqing Medical University, Chongqing, China; ^8^Department of Critical Care Medicine, The First Affiliated Hospital of Chongqing Medical and Pharmaceutical College, Chongqing, China; ^9^Department of Clinical Nutrition, The Affiliated Dazu Hospital of Chongqing Medical University, Chongqing, China

**Keywords:** Vanin-2, obesity, laparoscopic sleeve gastrectomy, inflammation, adipokines

## Abstract

**Objective:**

Excessive obesity can lead to dysfunction in adipose tissue, which contributes to the development of comorbidities associated with obesity, such as type 2 diabetes (T2D), cardiovascular and cerebrovascular disease, among others. Previous research has mainly focused on the Vanin family in systemic inflammatory diseases or predicting its role in tumor prognosis, while neglecting its role as a secretory protein in adipose tissue inflammation and metabolism. The objective of this study was to compare the changes in Vanin-2 levels in the circulating blood of normal and obese individuals, and to assess its correlation with inflammatory factors *in vivo*. Furthermore, the study aimed to systematically evaluate its effectiveness in human weight loss surgery.

**Methods:**

Serum concentrations of Vanin-2 and inflammatory indicators were measured in 518 volunteers. Furthermore, the concentrations of Vanin-2 were measured both before and after weight loss through a dietetic program or laparoscopic sleeve gastrectomy (LSG). Additionally, we assessed the levels of insulin, adiponectin, and inflammation-related factors. The hormonal profile and changes in body weight were evaluated at baseline and 3 months after surgery.

**Results:**

Serum levels of Vanin-2 were found to be significantly increased in individuals with overweight/obesity (OW/OB) group (controls 438.98 ± 72.44, OW/OB 530.89 ± 79.39 ug/L; *p* < 0.001). These increased levels were associated with IL-18, BMI, FAT%, and HOMA-IR. However, levels of Vanin-2 remained unchanged after conventional dietary treatment. On the other hand, weight loss induced by LSG resulted in a significant decrease in Vanin-2 concentrations from 586.44 ± 48.84 to 477.67 ± 30.27 ug/L (*p* < 0.001), and this decrease was associated with the Vanin-2 concentrations observed before the operation.

**Conclusion:**

Serum Vanin-2 is a highly effective biomarker for assessing adipose tissue inflammation in obesity and has the potential to serve as a predictor of bariatric surgery outcomes.

## Introduction

Obesity and its associated metabolic diseases have emerged as significant health concerns. The escalating prevalence of obesity is expected to contribute to a higher incidence of conditions such as hypertension, diabetes, hypercholesterolemia, chronic obstructive pulmonary disease, and even cancer. These conditions pose a serious threat to human health and well-being (1–3). However, the current methods for preventing or treating obesity have not yielded satisfactory results. Therefore, it is crucial to elucidate the causes and mechanisms of obesity to identify new targets for effective fat loss. Adipose tissue serves as a dynamic and active endocrine organ that regulates various physiological and pathological processes in the body. It influences hemostasis, blood pressure, lipid and glucose metabolism, inflammation, and atherosclerosis by secreting a range of adipokines (4). In obesity, adipose tissue secretes a large number of proinflammatory factors, while the secretion of anti-inflammatory factors such as adiponectin (5) is reduced and leptin resistance (6) This imbalance leads to chronic low-grade inflammation in adipose tissue and overall, causing obesity and related metabolic diseases. Therefore, the discovery and study of novel anti-inflammatory adipokines will provide new ideas for improving obesity-induced fat dysfunction and obesity-related metabolic diseases.

The human Vanin gene family consists of Vanin-1 (VNN 1), Vanin-2 (VNN 2), and Vanin-3 (VNN 3) (7). Vanin is conserved across species, but human Vanin-3 was recently classified as a pseudogene, meaning it does not encode the expected Vanin-3 protein (8). Human Vanin-2 is expressed in almost all tissues, with the highest expression observed in the blood, particularly in neutrophils (7). Based on the similarity in expression pattern and protein structure, it is possible that mouse Vanin-3 has a functional correspondence to human Vanin-2 (8). The Vanin gene family encodes an enzyme known as pantetheinase, which hydrolyzes mercaptoacetamine, an intermediate product of the coenzyme A degradation pathway. coenzyme A (CoA) is a cofactor involved in various biological processes, including fatty acid synthesis, steroid hormone production, and pyruvate oxidation for the citric acid cycle (8). The hydrolysis of mercaptoylamine by Vanin produces pantothenic acid (vitamin B5) and cysteamine, an effective antioxidant (9). Cysteamine, a potent antioxidant, has been found to protect islet cells and type 1 diabetes from oxidative stress (10, 11). The Vanin family genes and the metabolite cysteamine also play significant roles in inflammatory and oxidative responses. Recent studies have associated high gene expression of Vanin with diseases like steatosis (12) and diabetes (13). However, Vanin-1 is the most extensively studied member of this family. Previous research has demonstrated the crucial role of Vanin-1 in regulating lipolysis and obesity through PPAR-α and PPAR-γ (14). Furthermore, there is evidence suggesting a correlation between Vanin-1 levels in obese patients and their blood glucose, inflammatory markers, and oxidative stress (15). In a study by Chen et al. (16), Vanin-1 was shown to activate liver gluconeogenesis in mice, indicating that the Vanin family could be a potential therapeutic target for adipose tissue and metabolic diseases. Targeting the enzymatic activity of panaminase using specific inhibitors may lead to the development of a new class of anti-inflammatory antioxidants or even weight loss drugs (17). However, some studies have found that *vnn1* KO mice (whole-body knockout mice of the VNN 1 gene) do not exhibit any noticeable an obvious spontaneous phenotype (18). This implies that other members of the Vanin family might have a more significant role or a synergistic effect.

The regulatory effects of Vanin-2, a member of the human Vanin family, both individually and collectively, are not yet understood. This study aims to explore the expression patterns and potential mechanisms of Vanin-2 in individuals with obesity and insulin resistance. We hypothesized that serum Vanin-2 concentrations would increase with the severity of obesity. To test this hypothesis, we also examined the impact of modified dietary habits and weight loss surgery on serum Vanin-2 levels.

## Research design and methods

### Study population and ethics statement

**Figure 1:**
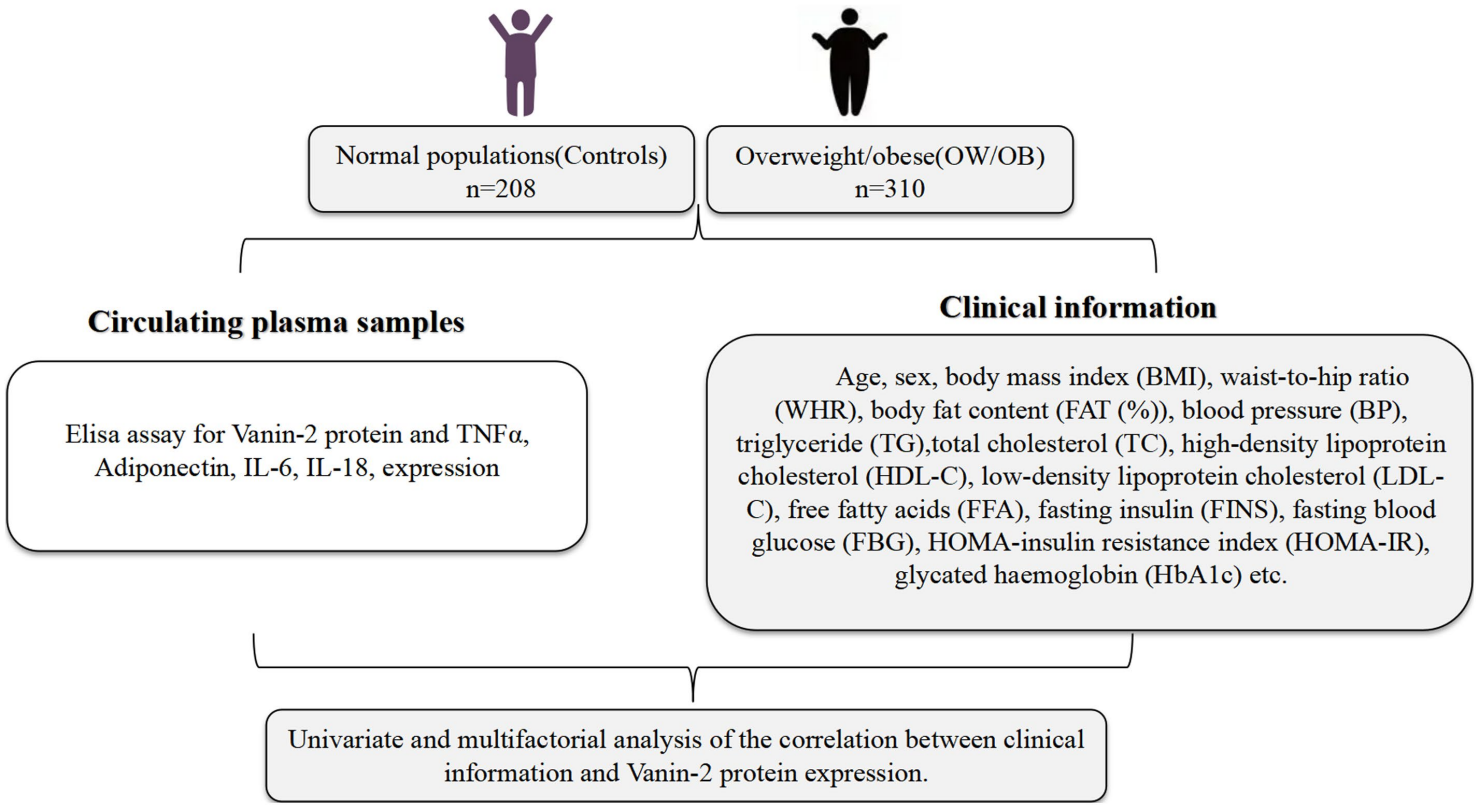
Clinical experimental design.

The study recruited a total of 518 participants, aged between 18 and 70, from the Dazu Hospital affiliated to Chongqing Medical University (Figure 1). Out of these, 310 were classified as obese individuals and 208 as healthy controls. The participants were divided into two groups based on their body mass index (BMI) according to the Chinese Diabetes Society criteria (CDS guideline 2017) (19). The first group consisted of individuals with a normal BMI (18 < BMI < 25 kg/m^2^), while the second group included those who were overweight or obese (BMI ≥25 kg/m^2^). Exclusion criteria for the study included well-diagnosed diabetes, coronary heart disease (CHD), chronic renal or hepatic diseases, autoimmune diseases, endocrine disease, infectious disease, and cancer. The normal control group consisted of individuals who had a normal physical examination, were from the community population or were university students, and had no metabolic or other diseases. Additionally, they had normal liver and kidney function. Participants were also required to have not used medications that alter glucose and lipid metabolism in the past 3 months.

**Figure 2:**
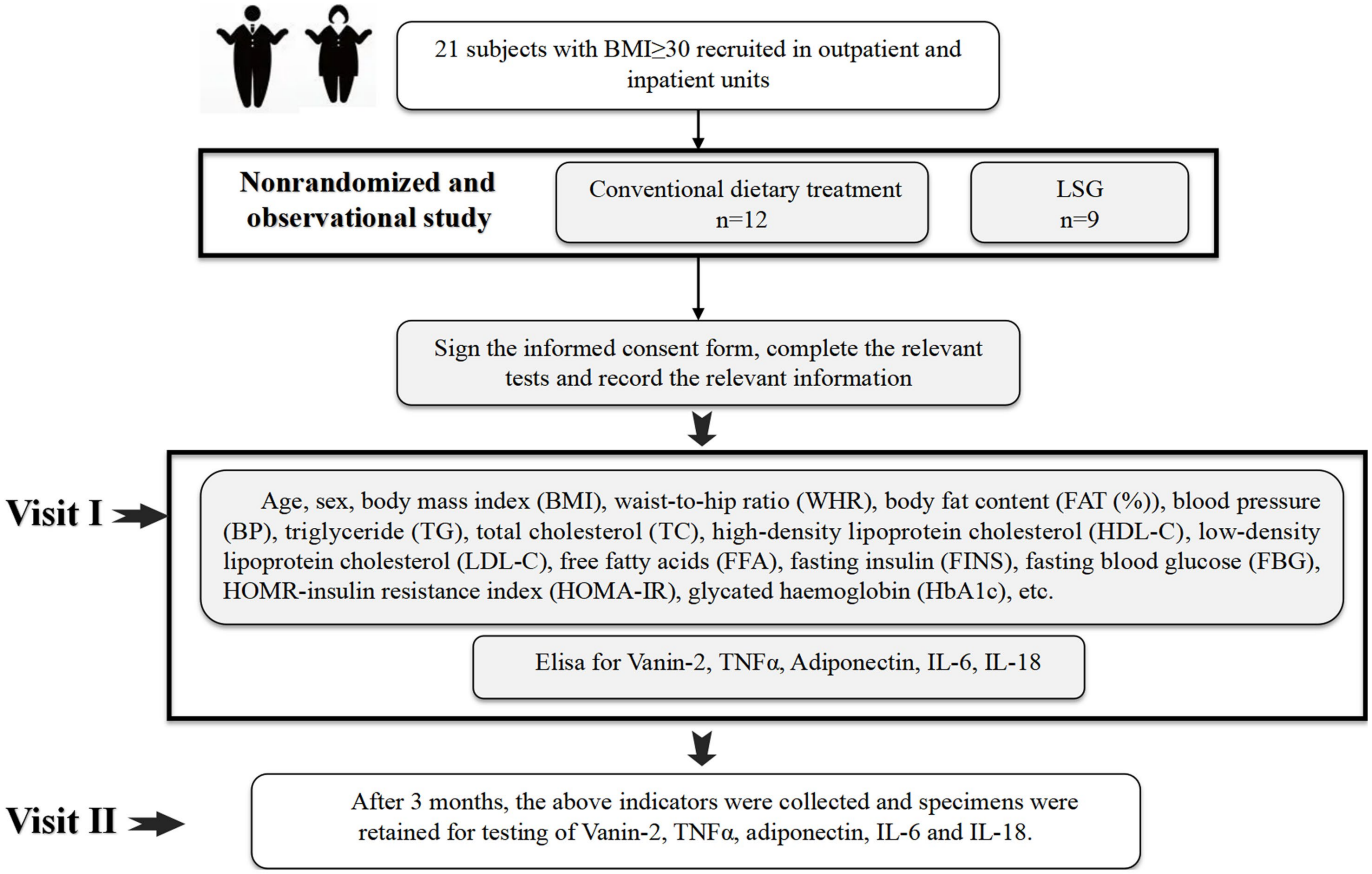
Clinical experimental design.

In addition to the randomized controlled trial, we also conducted a non-randomized controlled observational clinical trial (Figure 2). We recruited both outpatient and inpatient patients aged 18–65 years with a body mass index (BMI) of ≥30 kg/m^2^ (*n* = 21) who were eligible for bariatric surgery. After discussing with their doctors, 9 patients agreed to undergo LSG weight loss surgery, while the remaining patients received diet and lifestyle intervention for weight loss under the guidance of professional doctors at our hospital’s obesity clinic. Subject information was collected before the intervention, and a second round of data collection was conducted 3 months after the intervention. All assessments were carried out by the same team of researchers, physicians, and physiotherapists. Exclusion criteria included clinical and/or mental instability, unrealistic post-operative target weight and/or expectations, cancer, pregnancy, lactation, or planned pregnancy within 2 years, among others.

We obtained written voluntary informed consent from all subjects before they participated in the study. The current study followed the principles of the Declaration of Helsinki (20) and was approved by the ethics committee of Chongqing Medical University, and was registered at www.chictr.org.cn (ChiCTR2300076642).

### The dietary or lifestyle interventions

In the dietary or lifestyle group, a diet plan was formulated by a nutritionist of department of Clinical Nutrition in the Affiliated Dazu hospital of Chongqing medical university. Based on each person’s target energy intake, the daily energy intake was reduced by 500–1,000 kcal, and finally, the daily energy intake of male patients with obesity was 1,200–4,000 kcal, and the daily energy intake of female patients with obesity was 1,000–1,200 kcal, of which carbohydrate accounts for 55%–60% of total daily energy, and fat accounts for 25%–30% of total daily energy. All patients in the control group were required to perform at least 150–200 min of aerobic exercise a week. Subsequent individual sessions (usually weekly) and group sessions with the case managers were designed to reinforce the behavioral changes.

### LSG

All LSG procedures were performed laparoscopically by an experienced bariatric surgeon. The patient was placed in the reverse Trendelenburg position and a stamped card was used. Gastrectomy was performed at a distance of 2–6 cm from the pylorus, guided by a 36F bougie. We utilized the Medtronic nail gun to perform a cut closed sleeve gastrectomy, followed by sectional reinforced suture for hemostasis and a leak test with methylene blue. If there were no complications, the patient was discharged 2 days after the surgery.

### Anthropometric and laboratory measurement

All subjects underwent measurements of weight and height to calculate the BMI (weight in kg/height in m^2^). Waist circumference (WC) was measured at the narrowest indentation between the 10th rib and the iliac crest. Waist-to-hip ratio (WHR) was calculated as waist circumference (WC) divided by hip circumference (HC). Blood pressure (BP) was measured using a manual sphygmomanometer while the patient was seated, with the reading representing the mean of multiple measurements. Body fat percentage (FAT) was measured using bioelectrical impedance (BIA-101; RJL Systems). Insulin resistance was assessed using the homoeostasis model assessment of insulin resistance (HOMA-IR), calculated using the following equations: HOMA-IR = [fasting insulin (FIns, mU/L) × fasting blood glucose (FBG, mM)]/22.5 (21). After a minimum of 8 h of overnight fasting, venous blood samples were collected from all subjects, and serum was separated and stored at −80°C for future use. Blood glucose and HbA1c were immediately measured using the glucose-oxidase method and anion-exchange HPLC, respectively. Lipid profile, fasting insulin (FIns), and free fatty acid (FFA) were examined as previously reported (22).

### OGTT

Oral glucose tolerance tests (OGTTs) (23) were conducted at 8: 00 am, after a fasting period of 10–12 h. All subjects received an administration of 75 g of glucose. Blood samples were taken at 0, 30, 60, and 120 min to measure blood glucose levels, insulin levels, and serum Vanin-2 concentrations.

### Cytokine measurements

Place whole blood samples were collected at clinical laboratory of The Affiliated Dazu Hospital of Chongqing Medical University and at room temperature for 2 h or at 2°C–8°C overnight. Centrifuge for 20 min at 1000 × g and collect the supernatant to detect immediately or aliquot the supernatant and store it at −20°C or −80°C for future’s assay.

Human serum Vanin-2 kit (#3303, Chongqing Biospes Co., Ltd.) was based on sandwich enzyme-linked immune-sorbent assay technology. Vanin-2 antibody was precoated onto the 96-well plate. The biotin conjugated antibody was used as the detection antibody. The standards and pilot samples were added to the wells subsequently. After incubation, unbound conjugates were removed by wash buffer. Then, biotinylated detection antibody was added to bind with antigen conjugated on coated antibody. After washing off unbound conjugates, HRP-Streptavidin was added. After a third washing, TMB substrates were added to visualize HRP enzymatic reaction. TMB was catalyzed by HRP to produce a blue color product that turned yellow after adding acidic stop solution. Read the O.D. absorbance at 450 nm in a microplate reader. The concentration of target antigen in the sample is positively correlated with OD450 and can be calculated by plotting the standard curve (24).

Serum concentrations of TNF-α (Abcam, United States, ab181421) (25), IL-6 (Abcam, United States, ab178013) (26), IL-18 (Abcam, United States, ab215539) (27), and adiponectin (Abcam, United States, ab99968) (28) were measured using highly sensitive and specific ELISA kits according to the manufacturer’s instructions. These kits demonstrated no significant cross-reactivity. The intra- and interassay coefficients of variation (CVs) were <10 and <12% for Vanin-2, <2.5 and <3.1% for TNF-α, <2.1 and <2.4% for IL-6, <2.7 and <14.2% for IL-18, and <8 and <12% for Adipoq, respectively.

### Bioinformatics analysis

The STRING database (version 11.5) was used to search for information on VNN2/Vanin-2. The PPI network of VNN2/Vanin-2 was constructed based on the search results. Following the guidelines provided in the STRING manual, the first neighborhood of VNN2/Vanin-2 with an interaction score greater than 0.4 (medium confidence) was included. Additionally, KEGG enrichment and Gene Ontology analysis were conducted using the STRING database (*p* < 0.05).

### Statistical analysis

Statistical analyses were conducted using SPSS 19.0. The data were presented as mean ± SD or median with interquartile range. Non-normally distributed data, as determined by the Kolmogorov–Smirnov test, were logarithmically transformed prior to analysis. Independent student’s *t*-tests were used to compare the two groups. The association between Vanin-2 and other variables was analyzed using simple and multiple correlation coefficients. Binary logistic regression analyses were performed to examine the association between serum Vanin-2 and obesity. The sensitivity and specificity of Vanin-2 in predicting obesity were investigated using a receiver operating characteristic (ROC) curve created with SPSS 19.0. The sample size was calculated using the following equation: *n* = (*Z*_1−*α*/2_ *σ*/*εμ*)^2^ (*σ*, standard error; *μ*, mean; *Z*_1−*α*/2_ = 1.96, *α* = 0.05; and *ε* = 10%). *p* < 0.05 was considered significant compared to the control.

## Results

### General clinical and biochemical characteristics in the study population

Table 1 presents the clinical characteristics of the study population. The findings indicate that individuals with overweight/obesity (OW/OB) had significantly higher levels of body mass index (BMI), waist-to-hip ratio (WHR), FAT (%), systolic blood pressure (SBP), diastolic blood pressure (DBP), triglyceride (TG), free fatty acid (FFA), fasting blood glucose (FBG), 2 h blood glucose after glucose overload (2 h-BG), fasting insulin (FIns), 2 h insulin after glucose overload (2 h-Ins), HOMA-IR, the area under the curve for glucose (AUCg), the area under the curve for insulin (AUCi), and HbA1c, compared to the control group. Conversely, high-density lipoprotein cholesterol (HDL-C) levels were significantly lower in OW/OB subjects. Age, total cholesterol (TC), and low-density lipoprotein cholesterol (LDL-C) did not show significant differences between the control group and OW/OB patients (Table 1).

**Table 1:** Main clinical features and circulating Vanin-2 levels in the study population.

Variable	Controls (*n* = 208)	OW/OB (*n* = 310)	*p*-value
Age (years)	48 (37–57)	50 (42–55)	0.545
Female	141	156	—
BMI (kg/m^2^)	21.63 (20.67–24.33)	27.23 (26.12–28.37)	<0.001
WHR	0.81 ± 0.05	0.85 ± 0.07	<0.001
FAT (%)	27.45 ± 3.44	34.12 ± 4.09	<0.001
SBP (mmHg)	122 (108–131)	133 (126–141)	<0.001
DBP (mmHg)	76 (67–82)	87 (82–92)	<0.001
TG (mmol/L)	1.17 ± 0.44	1.93 ± 0.78	<0.001
TC (mmol/L)	4,71 ± 1.13	4.89 ± 1.32	0.126
HDL-C (mmol/L)	1.30 ± 0.30	1.23 ± 0.24	<0.01
LDL-C (mmol/L)	2.86 ± 0.84	2.98 ± 0.89	0.123
FFA (μmol/L)	0.45 ± 0.16	0.68 ± 0.12	<0.001
FBG (mmol/L)	5.28 ± 0.49	7.07 ± 1.25	<0.001
2 h-BG (mmol/L)	6.26 ± 0.59	12.68 ± 3.06	<0.001
FIns (mU/L)	9.65 ± 1.24	13.17 ± 1.45	<0.001
2 h-Ins (mU/L)	34.1 (32.4–35.9)	77.45 (72.58–80.63)	<0.001
HOMA-IR	2.27 ± 0.38	4.17 ± 0.95	<0.001
AUCg (mmol × h/L)	13.95 (13.53–14.40)	30.83 (28.21–32.85)	<0.001
AUGi (mU × h/L)	65.00 (63.59–66.24)	134.91 (131.00–137.59)	<0.001
HbA1c (%)	5.12 ± 0.63	8.42 ± 1.81	<0.001
Vanin-2 (ug/L)	438.98 ± 72.44	530.89 ± 79.39	<0.001
Adiponectin (mg/L)	41.64 ± 15.91	32.60 ± 11.06	<0.001
TNF-α (ng/L)	39.47 ± 9.45	49.50 ± 12.38	<0.001
IL-6 (ng/L)	6.49 ± 23.52	8.77 ± 31.69	<0.001
IL-18 (ng/L)	2.70 ± 0.85	3.63 ± 0.68	<0.001

BMI, body mass index; WHR, waist-to-hip ratio; SBP, systolic blood pressure; DBP, diastolic blood pressure; TG, triglyceride; TC, total cholesterol; HDL-C, high-density lipoprotein cholesterol; LDL-C, low-density lipoprotein cholesterol; FFA, free fatty acid; FBG, fasting blood glucose; 2 h-BG, 2 h blood glucose after glucose overload; FIns, fasting insulin; 2 h-Ins, 2 h insulin after glucose overload; HOMA-IR: HOMA-insulin resistance index; AUCg, the area under the curve for glucose; AUCi, the area under the curve for insulin; HbA1c: glycosylated hemoglobin; values were given as means ± SD or median (interquartile range).

### Serum Vanin-2 concentrations are elevated in patients with obesity

**Figure 3:**
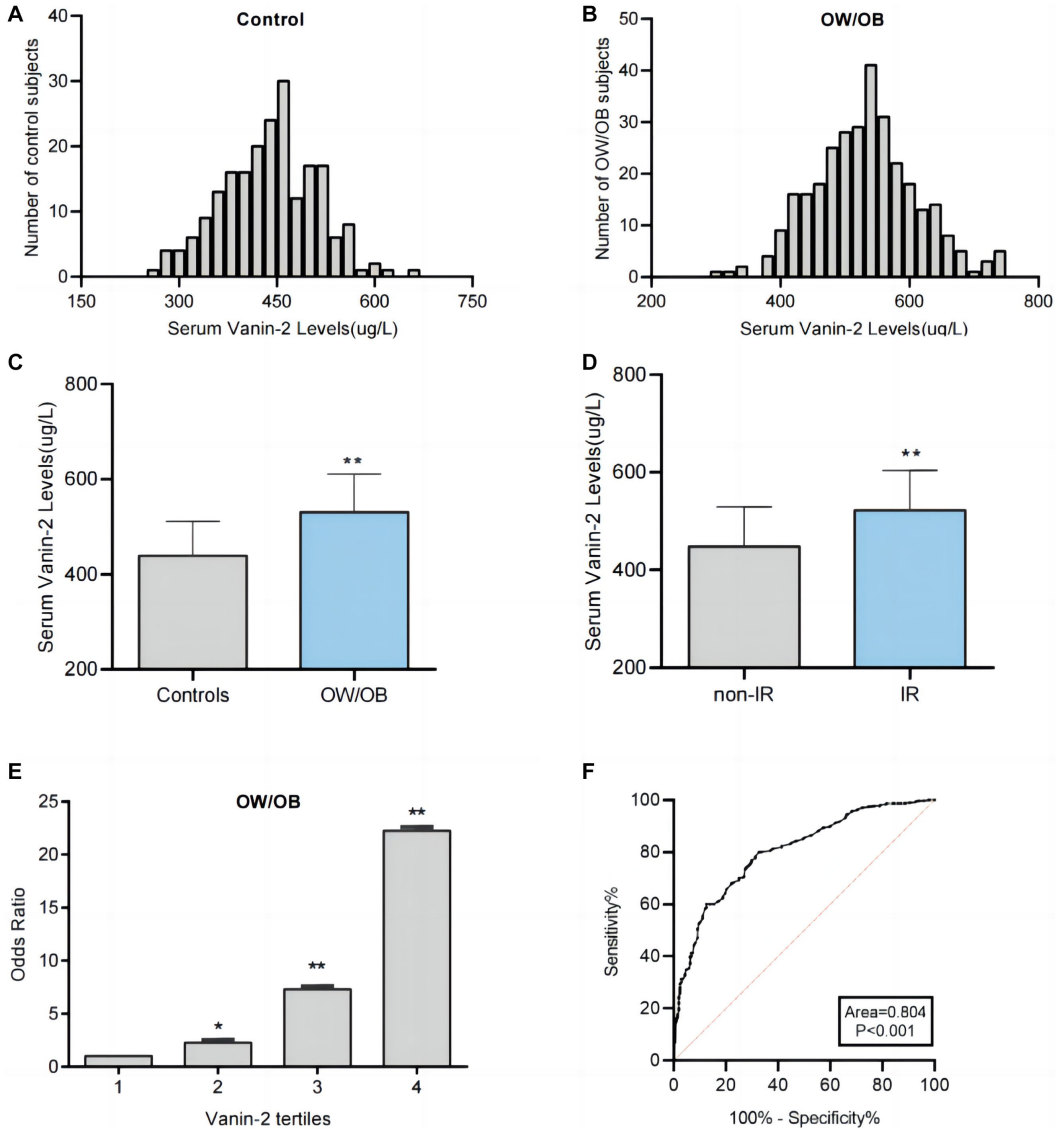
Analysis of serum Vanin-2 levels by different statistical approaches. **(A)** Distribution range of serum Vanin-2 concentration in the controls individuals. **(B)** Distribution range of serum Vanin-2 concentration in the OW/OB individuals. **(C)** Serum Vanin-2 levels in controls and OW/OB. **(D)** Serum Vanin-2 levels in non-insulin resistance (non-IR) and IR. **(E)** The odds ratio of having OW/OB in different quartiles of serum Vanin-2. **(F)** ROC curve analyses for the prediction of OW/OB. ^*^p < 0.05 and ^**^p < 0.01.

To investigate the relationship between Vanin-2 and OW/OB, we measured the levels of serum adiponectin, TNF-α, IL-6, IL-18, and Vanin-2 in the study population. Initially, we analyzed the distribution range of circulating Vanin-2 concentrations in all study populations. We observed that the Vanin-2 concentration range in normal controls was primarily 280–620 ug/L, while in OW/OB individuals, it was 320–720 ug/L (Figures 3A,B). Notably, patients with OW/OB exhibited significantly lower serum adiponectin levels, but higher Vanin-2, TNF-α, IL-6, and IL-18 levels compared to normal subjects (Table 1 and Figure 3C). And patients with IR exhibited significantly higher Vanin-2 levels compared to no-IR subjects (Figure 3D). These findings further support the association between Vanin-2, hypoadiponectin, and markers of inflammation in OW/OB populations. The study population was divided into four tertiles based on Vanin-2 concentration: tertile 1 (≤435 ug/L), tertile 2 (435–495 ug/L), tertile 3 (495–552 ug/L), and tertile 4 (>552 ug/L). Logistic regression analysis was used to calculate the odds of developing OW/OB for each tertile. The odds ratios for developing OW/OB were 2.259 (95% CI 1.353–3.771), 7.312 (95% CI 4.222–12.663), and 22.235 (95% CI 11.169–44.264) when Vanin-2 levels were in tertile 2, tertile 3, and tertile 4, respectively, compared to tertile 1 (both *p* < 0.01; Figure 3E and Table 2).

**Table 2:** Association of different concentrations of serum Vanin-2 level in OW/OB subjects.

group	Vanin-2	Univariable		
		OR	95% CI	*p*
OW/OB	Vanin-2, categorical (Tertile 1 as reference)	1	—	—
	Tertile 2	2.259	1.353–3.771	0.002
	Tertile 3	7.312	4.222–12.663	<0.001
	Tertile 4	22.235	11.169–44.264	<0.001

OR: odd ratio; 95% CI, 95% confidence interval.

To assess the predictive value of Vanin-2 for OW/OB, receiver operating characteristic (ROC) curve analysis was performed. The ROC curves of circulating Vanin-2 indicated an area under the curve (AUC) of 0.804, with a sensitivity of 80% and specificity of 68.3% for OW/OB (Figure 3F). The optimal cut-off value for serum Vanin-2 to predict OW/OB was determined to be 467.5 ug/L.

### Relationships between anthropometric and biochemical parameters and circulating Vanin-2

Table 3 presents the correlation analysis of Vanin-2 with various variables, including adiponectin, TNF-α, IL-6, and IL-18. Our findings indicate that circulating levels of Vanin-2 exhibit a significant positive correlation with age, BMI, WHR, FAT (%), BP, TG, FFA, FBG, FIns, HOMA-IR, HbA1c, TNF-α, IL-6, and IL-18, while showing a negative correlation with adiponectin (Table 3).

**Table 3:** Linear regression analysis of variables associated with serum Vanin-2 levels in all study population.

Variable	Simple		Multiple	
	*r*	*p*	*b*	*p*
Age (years)	0.006	0.886		
BMI (kg/m^2^)	0.412	<0.01	0.201	<0.001
WHR	0.207	<0.01		
FAT (%)	0.344	<0.01	0.139	<0.01
SBP (mmHg)	0.248	<0.01		
DBP (mmHg)	0.216	<0.01		
TG (mmol/L)	0.242	<0.01		
TC (mmol/L)	0.053	0.227		
HDL-C (mmol/L)	−0.027	0.54		
LDL-C (mmol/L)	0.063	0.155		
FFA (μmol/L)	0.286	<0.01		
FBG (mmol/L)	0.332	<0.01		
FIns (mU/L)	0.372	<0.01		
HOMA-IR	0.377	<0.01	0.144	<0.01
HbA1c (%)	0.339	<0.01		
Adiponectin (mg/L)	−0.125	<0.01		
TNF-α (ng/L)	0.225	<0.01		
IL-6 (ng/L)	0.192	<0.01		
IL-18 (ng/L)	0.297	<0.01	0.118	<0.01

In Pearson correlation analysis, values included for analysis were age, BMI, WHR, Fat%, SBP, DBP, TG, TC, HDL-C, LDL-C, FFA, FBG, FIns, HOMA-IR, HbA1c (%), adiponectin, TNF-α, IL-6, IL-18. Multiple stepwise regression showing variables with significant independent associations with serum Vanin-2.

**Figure 4:**
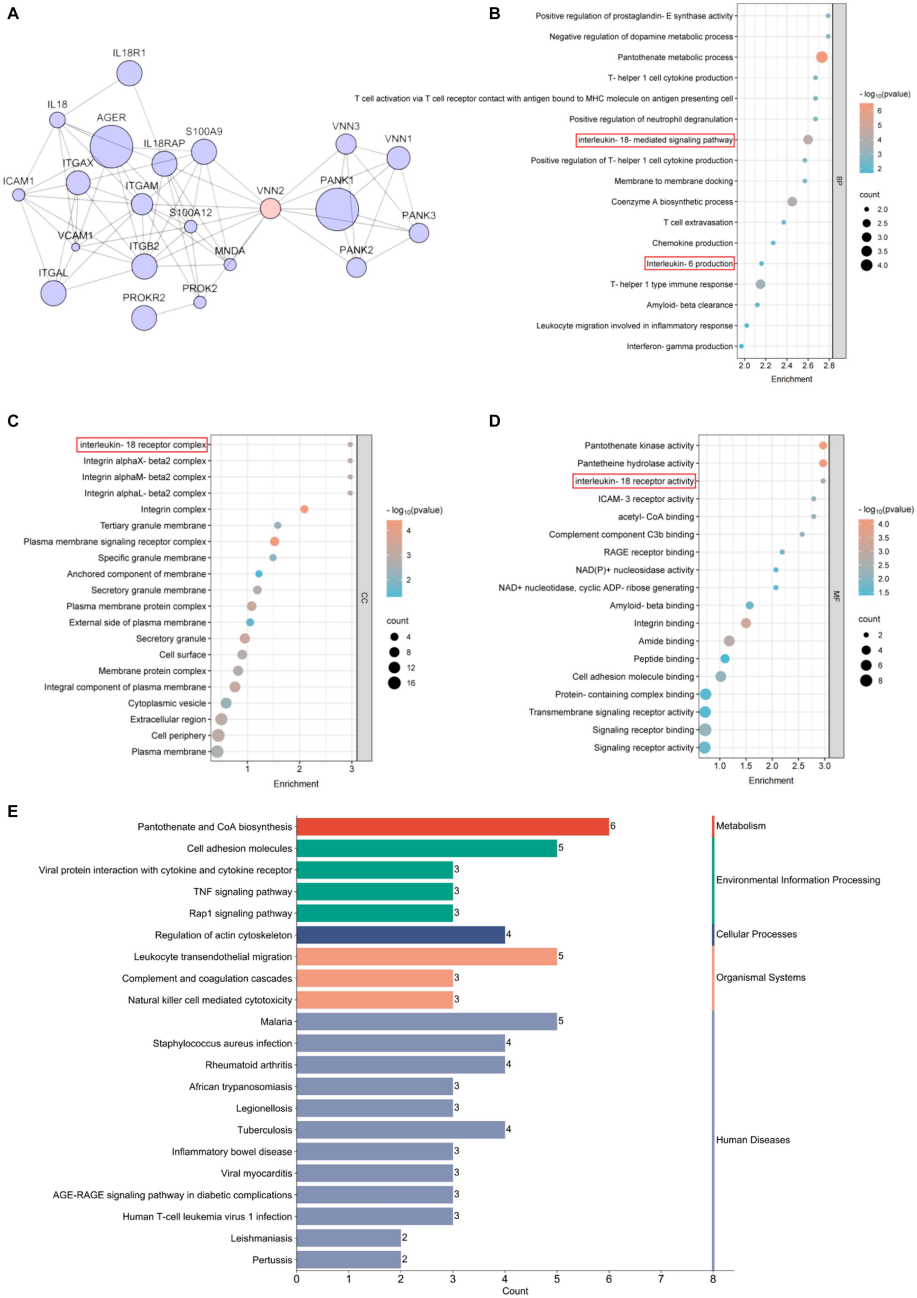
Bioinformatics analysis related to Vanin-2. **(A)** PPI network. **(B)** Biological process, BP. **(C)** Cellular component, CC. **(D)** Molecular function, MF. **(E)** KEGG enrichment.

To elucidate the molecular mechanisms by circle Vanin-2, we used the Search Tool for the Retrieval of Interacting Genes/Proteins (STRING) database to construct a protein and protein interaction (PPI) network. Twenty proteins residing in the first neighborhood of VNN2/Vanin-2 were identified, including IL-18 and IL-18r1 (Figure 4A). Ontology (GO) analysis in the biological process (BP) category revealed that the PPIs related to VNN2/Vanin-2 were involved in IL-18 mediated signaling pathway (Figure 4B). GO analysis in the cellular component (CC) category revealed IL-18 receptor complex (Figure 4C). GO analysis in the molecular function (MF) category revealed the involvement of a IL-18 receptor activity (Figure 4D). Kyoto Encyclopedia of Genes and Genomes (KEGG) pathway analysis indicated a strong correlation between VNN2/Vanin-2 PPIs and pantothenate and CoA biosynthesis, and the inflammation-related pathways (Figure 4E).

**Figure 5:**
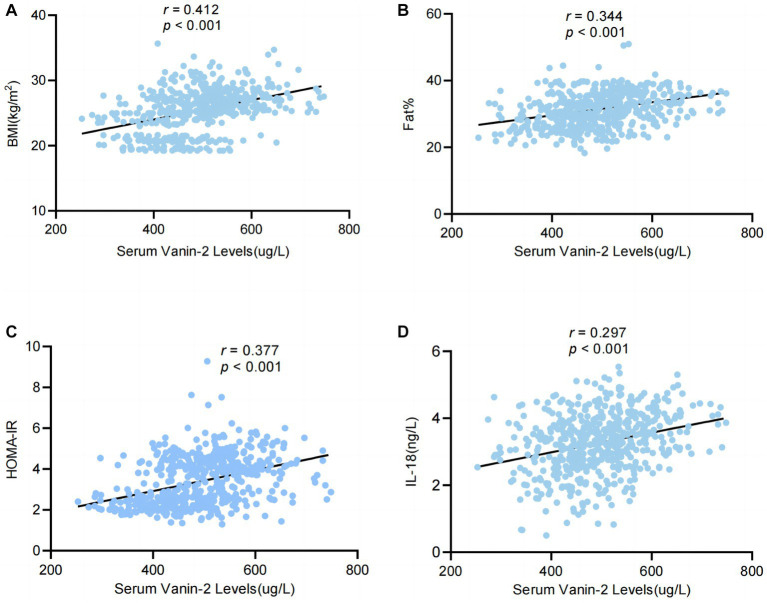
Serum levels of Vanin-2 increase with obesity in relation to inflammation of adipose tissue. **(A)** Serum Vanin-2 correlates with BMI **(B)** serum Vanin-2 correlates with FAT%, HOMA-IR **(C)** and circulating concentrations of IL-18 **(D)**. Pearson’s correlation coefficient (*r*) and *p*-values are indicated.

Through multivariate linear regression analyses, we identified BMI, FAT (%), HOMA-IR, and IL-18 as independent influential factors for circulating Vanin-2 (Figures 5A–D and Table 3). The regression equation is as follows: *Y*_Vanin-2_ = 200.30 + 5.57*X*_BMI_ + 2.46*X*_FAT(%)_ + 10.57*X*_HOMA-IR_ + 11.90*X*_IL-18_ (*R*^2^ = 0.469, *p* < 0.05) (Table 2). According to Table 3, logistic regression analysis demonstrated a significant correlation between Vanin-2 concentrations and OW/OB, even after controlling for anthropometric variables, age, gender, IL-18, and other factors (Table 4).

**Table 4:** Association of serum Vanin-2 with overweight/obese in fully adjusted models.

Model adjustment	OW/OB		
	OR	95% CI	*p*
Age	1.016	1.013–1.019	<0.001
Age, gender	1.016	1.013–1.020	<0.001
Age, gender, WHR	1.017	1.014–1.021	<0.001
Age, gender, WHR, FAT (%)	1.016	1.012–1.021	<0.001
Age, gender, WHR, FAT (%), BP	1.015	1.010–1.020	<0.001
Age, gender, WHR, FAT (%), BP, TG	1.016	1.010–1.023	<0.001
Age, gender, WHR, FAT (%), BP, TG, FBG	1.013	1.004–1.023	0.004
Age, gender, WHR, FAT (%), BP, TG, FBG, FIns	1.020	1.005–1.036	0.011
Age, gender, WHR, FAT (%), BP, TG, FBG, FIns, HOMA-IR	1.017	1.002–1.031	0.026
Age, gender, WHR, FAT (%), BP, TG, FBG, FIns, HOMA-IR, IL-18	1.017	1.002–1.033	0.025

Results of binary logistic regression analysis are presented. 95% CI, confidence interval; OR, odds ratio. Data were pooled to calculate logistic regression.

### Circulating Vanin-2 concentration in OGTT

**Figure 6:**
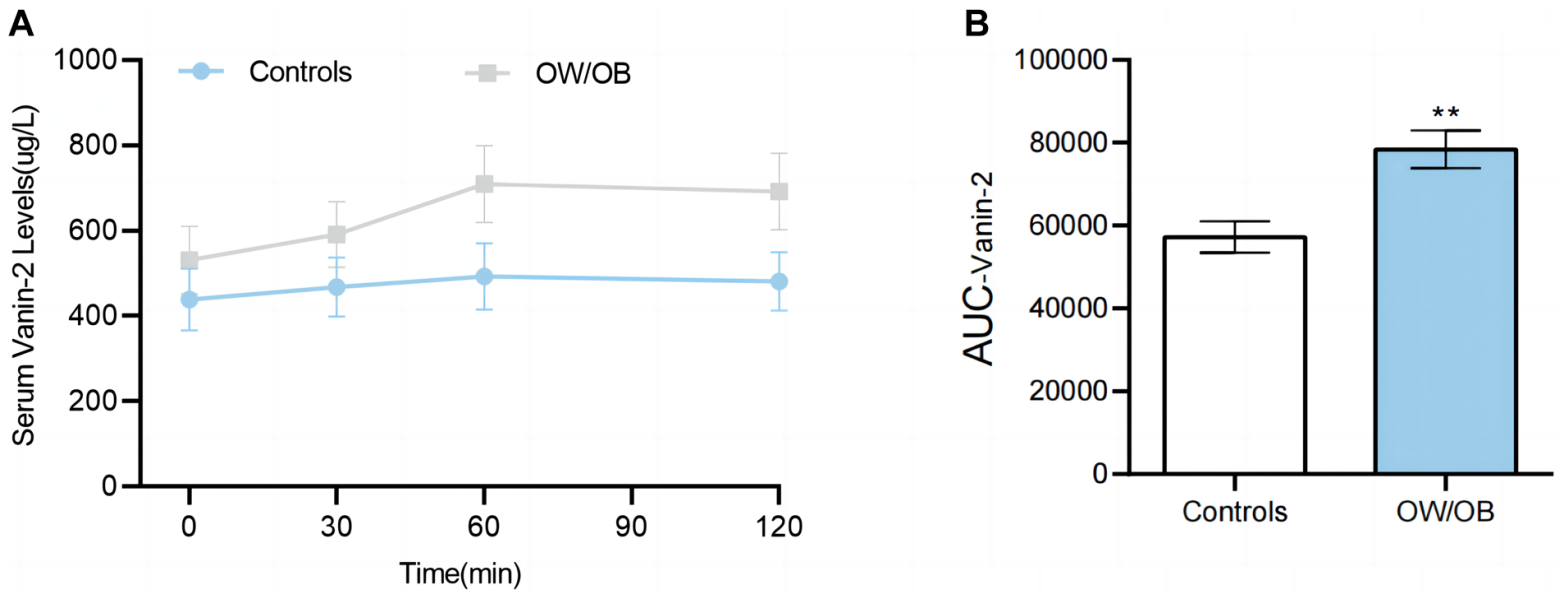
Circulating Vanin-2 concentration in OGTT **(A)** Vanin-2 concentration during the OGTT. **(B)** AUC_Vanin-2_ during the OGTT. Data are means ± SD or means ± SEM. ^*^p < 0.05 and ^**^p < 0.01.

To further investigate the relationship between blood glucose and Vanin-2, we performed OGTT experiments on both OW/OB and healthy individuals. The findings revealed that the levels of serum Vanin-2 remained relatively stable during the OGTT in both normal controls and OW/OB individuals (Figure 6A and Table 5). However, the AUC_Vanin-2_ in OW/OB patients was significantly higher compared to controls (Figure 6B). These results suggest that the concentration of circulating Vanin-2 is not influenced by blood glucose.

**Table 5:** Serum Vanin-2 levels in Controls and OW/OB subjects from minutes 0 to 120 min during OGTT.

Vanin-2 (ug/L)	0 min	30 min	60 min	120 min
Controls (*n* = 208)	438.98 ± 72.44	467.63 ± 69.83	493.15 ± 77.84	481.44 ± 68.14
OW/OB (*n* = 310)	530.89 ± 79.39	591.13 ± 77.11	709.85 ± 90.33	692.08 ± 89.63

Values were given as means ± SD. ^*^p < 0.05 and ^**^p < 0.01 compared with 0 min.

### Vanin-2 concentrations decrease following LSG but not after diet-induced weight loss

**Figure 7:**
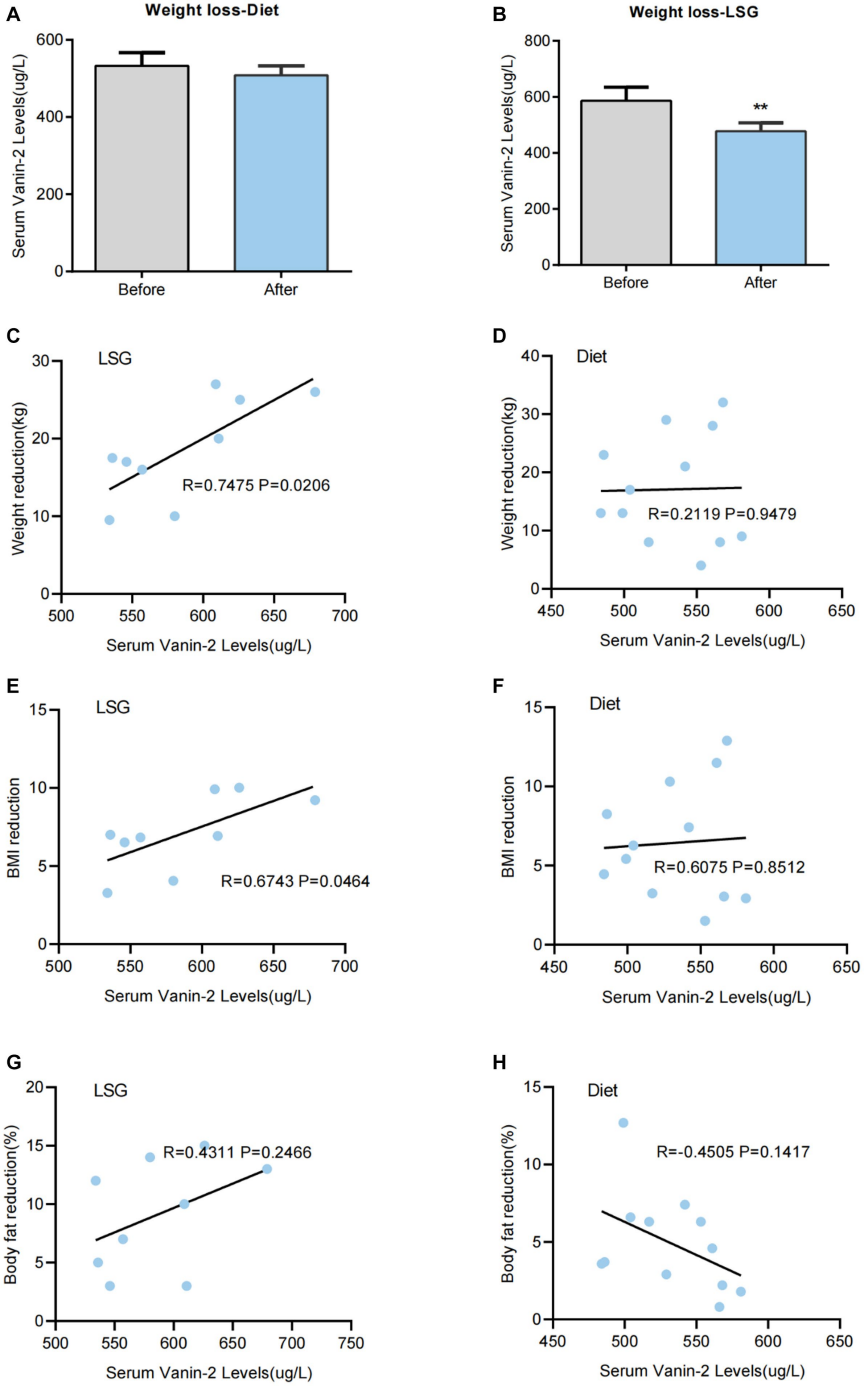
Serum Vanin-2 levels are changed after bariatric surgery-induced weight loss (WL), but not after diet-induced WL. **(A)** Effect of WL on serum Vanin-2 levels in patients with obesity following a conventional dietary intervention or **(B)** laparoscopic sleeve gastrectomy (LSG). Bars representing the mean and the pre- and post-intervention dots are shown. Differences between pre and post weight change were analyzed by paired two-tailed student’s *t*-tests. ^*^p < 0.05 and ^**^p < 0.01. Scatter diagrams showing the correlations between the serum of Vanin-2 before WL with the levels of weight reductions following a LSG **(C)** or conventional dietary intervention **(D)**. The correlations between the serum of Vanin-2 before WL with the BMI reductions following a LSG **(E)** or conventional dietary intervention **(F)**. The correlations between the serum of Vanin-2 before WL with the Body fat reductions following a LSG **(G)** or conventional dietary intervention **(H)**. Pearson’s correlation coefficient (*r*) and *p*-values are indicated.

To investigate the impact of changes in body adiposity on Vanin-2 levels, we measured serum concentrations (Figure 2) following WL due to either conventional diet treatment or bariatric surgery. After 3 months of conventional dietary treatment, the average weight reduction was 17.08 ± 7.93 kg (*p* < 0.001), with a reduction in BMI of 6.44 ± 3.03 (*p* < 0.001), a mean decrease in FAT% of 4.9 ± 2.46% (*p* < 0.01), and a decrease in WHR (*p* < 0.01). There were no significant changes in blood pressure, glucose, insulin concentration, triglycerides, adiponectin levels, or inflammation-related factors (Table 6). Diet-induced weight loss did not result in a significant reduction in circulating Vanin-2 concentration, which remained at 532.50 ± 34.06 pg/mL before and 508.17 ± 24.83 pg./mL after WL (*p* = 0.058; Figure 7A).

**Table 6:** Effect of weight loss in patients with obesity after conventional dietary treatment or LSG.

Variable	Conventional dietary treatment			LSG		
	Before WL	After WL	*p*	Before WL	After WL	*p*
Age (years)	27.25 ± 2.99	—	—	36.67 ± 12.18	—	—
Male/female	6/6	—	—	2/7	—	—
Body weight (kg)	92.69 ± 9.83	75.84 ± 4.89	<0.01	94 ± 8.67	75.33 ± 8.22	<0.01
BMI (kg/m^2^)	35.30 ± 3.26	28.86 ± 3.13	<0.01	35.85 ± 5.18	28.76 ± 4.82	<0.01
WHR	0.86 ± 0.02	0.82 ± 0.02	<0.01	0.87 ± 0.04	0.80 ± 0.04	<0.01
FAT (%)	37.39 ± 1.11	32.48 ± 2.71	<0.01	38.44 ± 3.44	29.54 ± 2.78	<0.01
SBP (mmHg)	134.50 ± 6.64	130.17 ± 8.40	0.175	137.11 ± 14.79	124.44 ± 9.93	<0.05
DBP (mmHg)	90.08 ± 5.33	88.50 ± 5.18	0.468	94.22 ± 12.42	81.67 ± 6.27	<0.05
TG (mmol/L)	1.92 ± 0.33	1.81 ± 0.26	0.396	1.95 ± 0.45	1.22 ± 0.31	<0.01
TC (mmol/L)	4.85 ± 0.45	4.66 ± 0.51	0.345	4.87 ± 0.57	4.54 ± 0.51	0.217
HDL-C (mmol/L)	1.22 ± 0.17	1.28 ± 0.14	0.306	1.22 ± 0.11	1.27 ± 0.07	0.245
LDL-C (mmol/L)	3.05 ± 0.34	2.83 ± 0.41	0.177	3.15 ± 0.44	2.74 ± 0.32	<0.05
FFA (μmol/L)	0.67 ± 0.07	0.63 ± 0.06	0.202	0.69 ± 0.09	0.54 ± 0.07	<0.01
FBG (mmol/L)	7.37 ± 0.79	7.07 ± 0.81	0.37	7.48 ± 2.28	5.46 ± 0.72	<0.05
2 h-BG (mmol/L)	12.61 ± 2.82	11.59 ± 2.66	0.373	12.78 ± 2.74	9.64 ± 1.08	<0.01
FIns (mU/L)	13.44 ± 1.15	12.67 ± 0.90	0.08	15.74 ± 1.20	10.36 ± 1.03	<0.01
2 h-Ins (mU/L)	74.03 ± 8.97	68.13 ± 7.62	0.097	80.76 ± 10.44	47.74 ± 8.89	<0.01
HOMA-IR	4.41 ± 0.64	3.99 ± 0.61	0.117	5.23 ± 1.59	2.52 ± 0.48	<0.01
HbA1c (%)	8.44 ± 0.72	7.97 ± 0.71	0.117	8.50 ± 0.98	5.82 ± 0.74	<0.01
Vanin-2 (ug/L)	532.50 ± 34.06	508.17 ± 24.83	0.058	586.44 ± 48.84	477.67 ± 30.27	<0.01
Adiponectin (mg/L)	31.55 ± 6.61	35.55 ± 5.36	0.118	30.40 ± 7.40	39.42 ± 5.53	<0.05
TNF-α (ng/L)	53.74 ± 8.40	47.13 ± 8.67	0.071	55.58 ± 10.39	42.77 ± 8.77	<0.05
IL-6 (ng/L)	8.74 ± 10.44	7.96 ± 9.38	0.064	9.04 ± 10.41	7.06 ± 7.56	<0.01
IL-18 (ng/L)	3.69 ± 0.45	3.38 ± 0.33	0.071	3.57 ± 0.34	2.84 ± 0.22	<0.01

LSG, laparoscopic sleeve gastrectomy; WL, weight loss; BMI, body mass index; WHR, waist-to-hip ratio; SBP, systolic blood pressure; DBP, diastolic blood pressure; TG, triglyceride; TC, total cholesterol; HDL-C, high-density lipoprotein cholesterol; LDL-C, low-density lipoprotein cholesterol; FFA, free fatty acid; FBG, fasting blood glucose; 2 h-BG, 2 h blood glucose after glucose overload; FIns, fasting insulin; 2 h-Ins, 2 h insulin after glucose overload; HOMA-IR, HOMA-insulin resistance index; HbA1c, glycosylated hemoglobin; values were given as means ± SD. Differences between after vs before WL were computed by two-tailed paired student’s *t*-tests.

Three months after LSG, patients experienced significant reductions in weight (18.67 ± 5.19 kg, *p* < 0.01; Figure 7B), BMI (7.09 ± 1.75, *p* < 0.01), and FAT% (16.7%, *p* < 0.01). They also showed significant improvements in glucose (*p* < 0.05), insulin (*p* < 0.01), HbA1c (*p* < 0.01), and HOMA-IR index (*p* < 0.01) (Table 6). LDL-C (*p* < 0.05), FFA (*p* < 0.01), and inflammatory markers (*p* < 0.05) were significantly improved. LSG-induced weight loss significantly reduced the circulating Vanin-2 concentration from 586.44 ± 48.84 to 477.67 ± 30.27 pg/mL (*p* < 0.001, Figure 7B). Vanin-2 levels were significantly associated with weight reduction (*r* = 0.7475, *p* = 0.0206, Figure 7C), or BMI reduction (*r* = 0.6743, *p* = 0.0464, Figure 7D), but not with body fat reduction (*r* = 0.4311, *p* = 0.2466, Figure 7E). However, pre-weight loss Vanin-2 levels were not correlated with weight reduction (*r* = 0.2119, *p* = 0.9479, Figure 7F), BMI reduction (*r* = 0.6075, *p* = 0.8512, Figure 7G), or body fat reduction (*r* = −0.4505, *p* = 0.1417, Figure 7H) after dietary treatment for weight loss.

## Discussion

**Figure 8:**
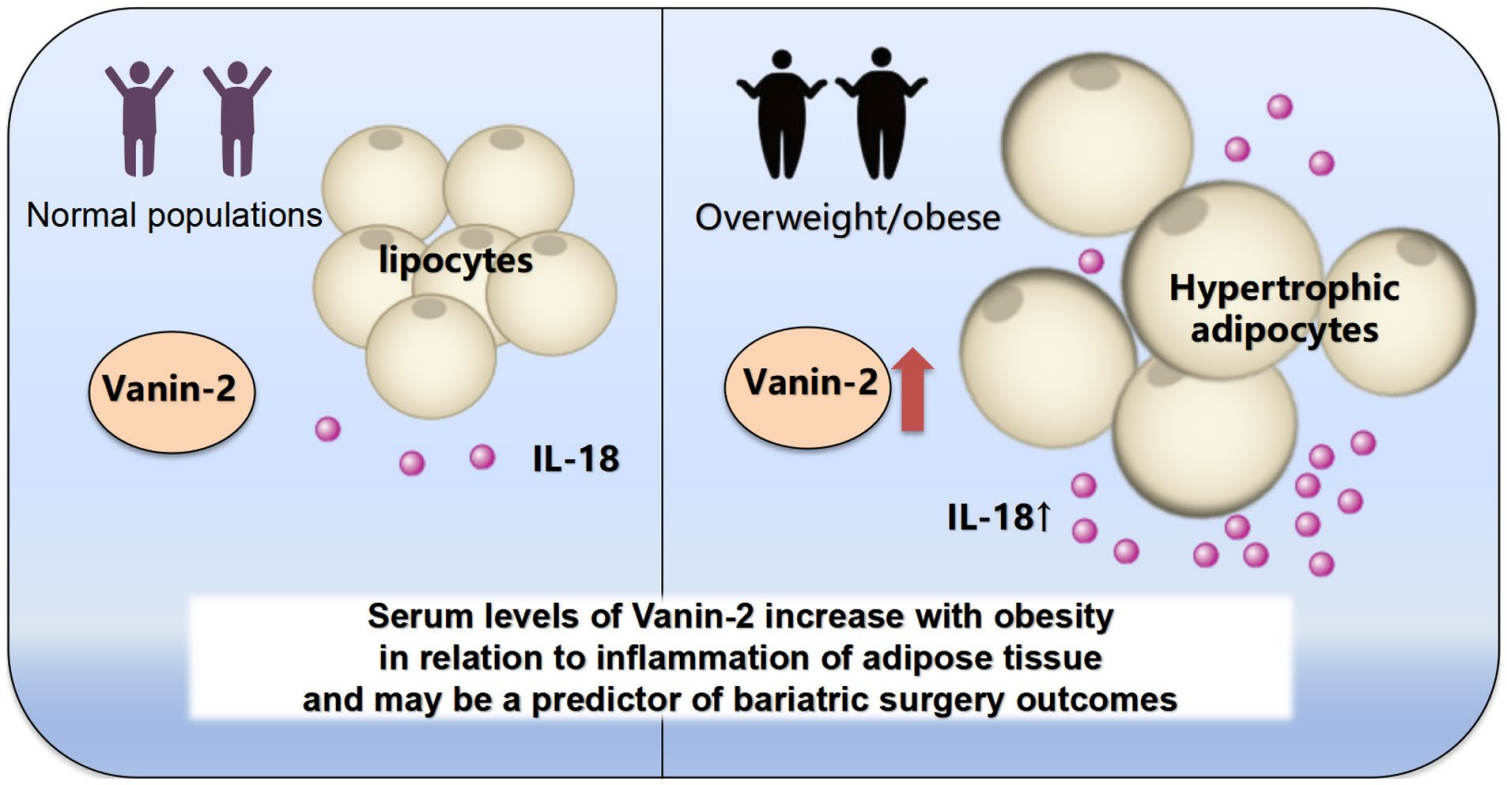
Schematic diagram.

The main findings of this study were as follows: (1) there was an increase in serum Vanin-2 concentrations in individuals with obesity and insulin resistance; (2) circulating Vanin-2 concentrations showed associations with IL-18, BMI, FAT%, and HOMA-IR; (3) weight loss following reduced weight surgery was linked to preoperative circulating Vanin-2 levels (Figure 8).

Studies have shown that adipose tissue plays a crucial role in the development and progression of obesity and related metabolic diseases. In cases of prolonged energy surplus, adipose tissue stores excess energy in the form of triglycerides through proliferation and hypertrophy. However, when fat cells expand and hypertrophy, it not only leads to glucose intolerance, hyperinsulinemia, type 2 diabetes, and cardiac metabolic complications, but also causes the deposition of lipids in peripheral tissues such as the liver and muscles (29). The development of insulin resistance (IR) is also closely linked to obesity (30). Compared to individuals with subcutaneous obesity, those with central obesity have a higher incidence of insulin resistance (31). Our research findings indicate a correlation between Vanin-2 levels and BMI and FAT%, suggesting that Vanin-2 levels are associated with the amount of adipose tissue. Furthermore, this study’s data show that serum Vanin-2 concentration is elevated in patients with insulin resistance. Additionally, Vanin-2 is associated with surrogate markers of insulin resistance, such as the HOMA-IR index, indicating that Vanin-2 levels are connected to insulin resistance, possibly due to increased adipose tissue.

Recent research has highlighted the significance of dynamic changes in adipocytes, immune cells, angiogenesis, and extracellular matrix remodeling in regulating the scalability and functional integrity of adipose tissue. In the context of obesity, macrophages are recruited and surround dying adipocytes, adopting an inflammatory phenotype. The number of adipocyte deaths is closely linked to the adverse effects of obesity, such as insulin resistance and hepatic steatosis (32). Vanin is an enzyme that breaks down mercaptoethylamine into panthenic acid (vitamin B5) and cysteamine, which is a potent antioxidant (8). Apart from its role in coenzyme A metabolism, the Vanin family genes and the metabolite cysteamine also play a significant role in inflammatory and oxidative responses. Serum levels of Vanin-1 have been associated with hepatic lipoplasia and have been considered as a non-invasive inflammatory marker for non-alcoholic steatohepatitis (12). Our study data revealed a positive correlation between Vanin-2 levels and Interleukin-18 (IL-18). IL-18, a member of the IL-1I cytokine superfamily, has pleiotropic functions (33, 34). It not only regulates immune cell communication but also plays a crucial role in metabolic control in normal physiology, obesity, diabetes, and related liver diseases (35–38). Studies have shown that endogenous IL-18 not only suppresses appetite but also promotes energy expenditure and lipid oxidation, which are involved in regulating body weight homeostasis and increasing fat mass (39–41). Therefore, we hypothesize that Vanin-2 may promote IL-18 secretion to maintain weight homeostasis and prevent excessive weight gain in individuals with obesity and insulin resistance. In conclusion, while the exact function of Vanin-2 is yet to be determined, the data suggest that dysfunctional adipose tissue in obesity leads to increased expression and secretion of Vanin-2. Moreover, Vanin-2 may play a role in regulating obesity-related inflammation and contribute to the development of protective metabolic disorders associated with obesity. Further preclinical studies on Vanin-2 are needed to address this question.

Over the past few decades, various weight-loss interventions have been developed, including lifestyle and behavioral interventions (such as diet and exercise), anti-obesity drugs, endoscopic interventions, and surgical weight-loss procedures. Surgical weight-loss procedures have been proven to be effective in treating obesity, type 2 diabetes the metabolic inflammation over the years (42–44). However, the clinical effects of weight-loss surgery vary among different obese individuals. Currently, the main criteria for selecting surgical candidates are body mass index (BMI) and related complications. Although the surgery can be performed once the criteria are met, accurately predicting the postoperative outcomes remains a challenge (45). Addressing this primary issue would significantly reduce the risk of surgical failure. Therefore, one of the key objectives of this study is to effectively predict the efficacy of weight-loss surgery and minimize the risk of surgical failure.

If weight loss surgery does not achieve the desired effect, it is often attributed to the recipient’s lack of good dietary control and physical exercise. However, the main reason for surgical failure is actually individual genetic differences (45). Elionora Pena et al. discovered that individuals with brain-derived neurotrophic factor (BDNF) methionine (Met) mutations experienced better weight loss after bariatric surgery (46). Mul’s et al. (47) study also revealed that animals with melanocortin 4 receptor (MC4R) deficiencies had significant resistance to weight loss after bariatric surgery. While these studies confirmed genetic variations as a factor in the effectiveness of weight-loss surgery, mutations in these genes are not commonly observed in the general population (48). Therefore, further genetic-level research is required to predict the efficacy of weight-loss surgery. Additionally, studies have shown that weight loss after bariatric surgery leads to a decrease in blood IL-18 concentration (49–51), which aligns with our findings.

To investigate the relationship between Vanin-2 levels and weight loss, we examined the effects of different weight loss methods. We observed a decrease in Vanin-2 levels after conventional dietary treatment, but a significant decrease was found after bariatric surgery-induced weight loss. Therefore, it can be concluded that changes in body weight have a major impact on plasma Vanin-2 levels, while changes in adiposity have a lesser effect. The changes in serum Vanin-2 levels in obese patients may indicate alterations in adipose tissue homeostasis. In diet-induced weight loss studies, Vanin-2 levels were found to be associated with IL-18 levels, as well as with weight loss and reduced BMI in patients undergoing bariatric surgery. It has also been suggested that IL-18 can be considered as a dynamic marker of adipose tissue homeostasis (40). Moreover, this study revealed that preoperative circulating Vanin-2 levels were associated with weight loss after weight loss surgery, and circulating Vanin-2 was correlated with IL-18 levels. The assessment of preoperative circulating Vanin-2 levels could potentially serve as a screening tool for predicting weight loss failure in obese individuals after weight loss.

This report examines the changes in Vanin-2 protein expression in obese or insulin-resistant (IR) patients, which may be associated with alterations in glycemic and inflammatory markers. The findings suggest that in obese patients, adipocytes produce Vanin-2 in peripheral tissues expressing IL-18, thereby inhibiting lipolysis. This study is novel and may be the first of its kind, with ongoing research to investigate the relationship between Vanin-2 and adipose tissue inflammation.

The study also discovered that the expression of Vanin-2 protein in the bloodstream could potentially serve as a predictor for the outcomes of bariatric surgery. However, it is important to note that this conclusion requires further support from additional clinical cases.

In this report, the researchers explored the relationship between changes in Vanin-2 protein expression and obesity or insulin resistance (IR) in patients. They found that these changes could be linked to alterations in glycemic and inflammatory factors. Specifically, the study suggests that in obese patients, adipocytes in peripheral tissues produce Vanin-2, which in turn inhibits lipolysis by interacting with IL-18. This novel finding has not been previously documented and represents a significant contribution to the field. Ongoing studies are currently underway to investigate the association between Vanin-2 and inflammation of adipose tissue.

However, our study does have some limitations: (1) although the overall size of the study population is large, the number of patients after grouping is still relatively limited, which is a common feature in cohort studies. (2) It is important to note that our study was conducted only in the Chinese population, so caution should be exercised when applying these findings to other ethnic groups. (3) We mentioned in the previous literature that *vnn1* KO mice do not exhibit any noticeable an obvious spontaneous phenotype, suggesting that the confirmed functional loss of the Vanin-1 protein alone may not be sufficient to affect the entire organism. Therefore, it may be more crucial to investigate the entire Vanin gene family. Additionally, we focused solely on Vanin-2 in this study as human Vanin-3 has recently been classified as a pseudogene. In future studies, we plan to further explore the correlation and mechanism between the Vanin family and obesity by studying the relationship between Vanin-1 and Vanin-2 factors and obesity, and potentially constructing human gene knockout mice.

In addition, the study also found that the Vanin-2 protein expression in the blood circulation may be a predictor of bariatric surgery outcomes. And this conclusion needs to be supported by more clinical cases.

## Data availability statement

The raw data supporting the conclusions of this article will be made available by the authors, without undue reservation.

## Ethics statement

The studies involving humans were approved by Ethics Committee of Dazu Hospital of Chongqing Medical University. The studies were conducted in accordance with the local legislation and institutional requirements. The participants provided their written informed consent to participate in this study.

## Author contributions

SG: Data curation, Writing – original draft, Conceptualization, Project administration. DC: Data curation, Writing – review & editing. YW: Conceptualization, Data curation, Writing – original draft, Writing – review & editing. XY: Writing – original draft. DZ: Writing – original draft. XL: Writing – original draft. XZ: Writing – review & editing. CH: Writing – original draft. XY: Writing – original draft. XM: Writing – original draft. WH: Writing – review & editing. JX: Supervision, Writing – review & editing. SY: Supervision, Writing – review & editing.

## Glossary

BMI: body mass index
WHR: waist-to-hip ratio
SBP: systolic blood pressure
DBP: diastolic blood pressure
TG: triglyceride
TC: total cholesterol
HDL-C: high-density lipoprotein cholesterol
LDL-C: low-density lipoprotein cholesterol
FFA: free fatty acid
FBG: fasting blood glucose
2 h-BG: 2 h blood glucose after glucose overload
FIns: fasting insulin
2 h-Ins: 2 h insulin after glucose overload
HOMA-IR: HOMA-insulin resistance index
AUCg: the area under the curve for glucose
AUCi: the area under the curve for insulin
HbA1c: glycosylated hemoglobin
LSG: laparoscopic sleeve gastrectomy
WL: weight loss
